# A comparative study on early vision quality after implantation of refractive segmental and diffractive multifocal intraocular lens

**DOI:** 10.12669/pjms.36.7.3364

**Published:** 2020

**Authors:** Huifang Lian, Weisong Ma, Qiuhong Wei, Xiaoyong Yuan

**Affiliations:** 1 Huifang Lian, Department of Ophthalmology, Clinical College of Ophthalmology, Tianjin Medical University, Tianjin, P.R. China. Tianjin Eye Hospital, Tianjin Eye Institute, Tianjin Key Laboratory of Ophthalmology and Visual Science, Tianjin, 300020, P.R. China, Baoding First Central Hospital, Hebei, Baoding 071000, P. R. China; 2 Weisong Ma, Department of Orthopaedics, Baoding First Central Hospital, Hebei, Baoding First Central Hospital, Hebei, Baoding 071000, P. R. China; 3 Qiuhong Wei, Department of Ophthalmology, Baoding First Central Hospital, Hebei, Baoding 071000, P. R. China; 4 Xiaoyong Yuan, Clinical College of Ophthalmology, Tianjin Medical University, Tianjin, P.R. China. Tianjin Eye Hospital, Tianjin Eye Institute, Tianjin Key Laboratory of Ophthalmology and Visual Science, Tianjin, 300020, P.R. China

**Keywords:** Multifocal intraocular lens, Asymmetry, Vision quality, Contrast sensitivity, Aberration

## Abstract

**Objectives:**

To compare early subjective and objective vision quality of postoperative patients undergoing phacoemulsification cataract surgery combined with implantation of refractive segmental multifocal intraocular lens (MIOL) SBL-3 and apodized diffractive MIOL SN6AD1.

**Methods:**

As a prospective study, it enrolled 53 patients (53 eyes) to undergo phacoemulsification cataract surgery combined with MIOL implantation. According to differences in MIOL implanted, patients were divided into a SBL-3 group (25 eyes) and a SN6AD1 group (28 eyes). Ophthalmological evaluation included uncorrected (UDVA) and corrected (CDVA) distance visual acuities, uncorrected intermediate (UIVA) and near (UNVA) visual acuities, distance-corrected intermediate (DCIVA) and near (DCNVA)visual acuities and corrected near(CNVA) visual acuity, contrast sensitivity, modulation transfer function (MTF) and high order aberration (4 mm pupil diameter) at three months postoperatively. Moreover, a questionnaire survey was carried out to assess near spectacle independence, patient satisfaction and symptoms of visual disturbance.

**Results:**

At three months after surgery, UIVA and UNVA in the SBL-3 group are statistically significantly superior to those of the SN6AD1 group (P>0.05). There was statistical difference in contrast sensitivity at four spatial frequencies (3, 6, 12, 18cycles/degree) under mesopic conditions and mesopic conditions with glare (P>0.05). The total ocular high order aberration, coma and trefoil were statistically significantly larger in the SBL-3 group than in the SN6AD1 group with 4.0 mm pupil diameters (P>0.05). Statistical differences were found in the MTF at spatial frequencies of 5, 10 and 15 cycles/degree between the groups. There were no significant differences in spectacle independence, patient satisfaction and visual disturbance between the groups (P>0.05).

**Conclusions:**

Both the two multifocal intraocular lens provided an excellent level of quality of vision three months postoperatively. However, the application effect of SBL-3 MIOL is superior to that of SN6AD1 MIOL as far as intermediate vision, near vision and contrast sensitivity are concerned.

## INTRODUCTION

With continuous development of medical techniques, cataract surgery has changed from a traditional vision restoration into a refractive surgery. An increasing number of patients with cataract begin to pursue a full range of vision and high vision quality by virtue of a cataract surgery. In order to improve better intermediate and near vision, different types of multifocal IOL(MIOL), appear in clinics for the past few years. According to design principle of the optical surfaces, traditional MIOLs can be divided into diffractive, refractive and refractive-diffractive lens.^1^ To be specific, MIOL produce two or more foci by redistributing light entering eyes based on the diffraction or refraction principle of light, so as to improve intermediate and near visual acuity.^2^ In comparison with monofocal IOL, more postoperative visual disturbance phenomena take place accordingly after MIOL implantation, mainly including contrast sensitivity reduction, glare and halo.^3^

Recently, a novel MIOL SBL3 (Lenstec, Inc., Christ Church, Barbados) has been introduced into clinical practice. Designed based on a concept of rotational asymmetry, SBL3 contains two refracting sector surfaces. It has a distance section combined with a 3.00 D near vision segment in the anterior optic separated by a small wedge-shaped transition zone. Fewer transition zones between different power zones should lead to less energy loss and improved near vision quality and contrast sensitivity.^4^

The aim of the study was to compare the postoperative visual acuity, subjective and objective vision quality after refractive segmental MIOL SLB-3 and apodized diffractive MIOL SN6AD1(Alcon Laboratories Inc., Fort Worth, Texas, United States) implantation. This study can be used as a reference for clinical application of refractive segmental MIOL.

## METHODS

As a prospective study, a total of 53 patients (53 eyes) undergoing phacoemulsification cataract surgery combined with MIOL implantation were enrolled. All the patients were divided into the SBL-3 group and the SN6AD1 group according to different types of IOLs implanted. The inclusion criteria were as follows: (i) patients with age-related cataract; (ii) all patients have signed the informed consent of this study and completed relevant follow-up visits; (iii) patients meeting the following conditions: length of optic axis between 22mm and 25mm; preoperative corneal astigmatism less than 1.0D; with normal pupil size and normal intraocular pressure, (iv) MIOL implantation in a capsular bag and at the right position. The exclusion criteria were patients with a history of ocular trauma or intraocular surgery, glaucoma, active uveitis, fundus disease, lens dislocation, corneal disease or patients combined with a systemic disease that may affect visual acuity, including diabetes, hypertension and nephropathy. The general condition comparisons between two groups are comparable (P>0.05), (Table-I). Moreover, the study adhered to the tenets of the Declaration of Helsinki.

**Table-I:** Preoperative general data for patients in two groups.

Group	Number of eyes	Age	Number of male and female cases	Pupil diameter (mm)	Length of optic axis (mm)	Corneal curvature (D)	Corneal astigmatism (D)	IOL degree (D)
SBL-3	25	64.16±0. 88	13/12	3.85±0.10	23.20±0. 10	43.78±0.10	-0.80±0.04	20.91±0.28
SN6AD1	28	64.11±0. 93	15/13	3.84±0.11	23.16±0. 12	43.82±0.08	-0.71±0.05	20.84±0.26
t / χ^2^		0.040	0.013	0.072	0.262	0.273	1.408	0.178
*P* value		0.968	0.909	0.943	0.795	0.786	0.165	0.859

### Ethical Approval

The study was approved by the Institutional Ethics Committee of Baoding First Central Hospital, (Dated July 7, 2020) and written informed consent was obtained from all participants.

### Preoperative Examinations

Ocular examinations are performed in detail for all patients before the surgery, including keratometry, topography, and autorefraction (OPD-Scan II ARK-10000, Nidek Co., Ltd.), slitlamp evaluation, Goldmann tonometry and retinal optical coherence tomography (DRI-OCT, Topcon, Tokyo, Japan). Biometry performed with the IOL Master (Carl Zeiss Meditec AG) measured corneal curvature, anterior chamber depth, and axial length (AL) for IOL calculation. The refractive aim was emmetropia.

### Surgical Technique

All surgeries were performed by the same experienced surgeon with standardized phacoemulsification. The surgery was performed using topical anesthesia. A 2.8mm primary transparent cornea incision was placed on the steepest corneal meridian. A 5.00 mm anterior capsulorhexis was created and the MIOL implanted in the capsular bag. The SBL-3 MIOL was positioned inferiorly with slight nasal deviation. No adverse events occurred. Postoperatively, Tobramycin Dexamethasone and Pranoprofen Eye Drops should be both dosed four times daily for four weeks.

Postoperative assessments were performed 3 months after surgery. The routine examinations were performed to observe ocular wound healing conditions, inflammatory responses of the anterior chamber, pupil conditions and the position of IOL.

Visual acuities were evaluated with logarithm of the minimum angle of resolution (logMAR) charts for distance (5 m) and with Radner reading charts for intermediate and near vision(80 cm and 40 cm). Evaluated were the uncorrected (UDVA) and corrected (CDVA) distance visual acuities, uncorrected intermediate (UIVA) and near (UNVA) visual acuities, distance-corrected intermediate (DCIVA) and near (DCNVA)visual acuities and corrected near(CNVA) visual acuity.

The same experienced optometrist made use of a CSV-1000E contrast sensitivity device to check contrast sensitivity of patients with best corrected visual acuity at four spatial frequencies (3, 6, 12 and 18 cycles/degree) under mesopic conditions (3cd/m^2^) or mesopic conditions with glare (28Lx).

The ocular total high order aberration, coma, trefoil, and spherical aberrations as well as the MTF values at 5, 10, 15, 20, 25, and 30 cycles/degree were recorded using iTrace (Tracey Technologies, Houston, Texas, United States) with 4.0 mm pupil diameters.

Questionnaires were produced by referring to Quality-of-Life Survey for Patients after MOIL Implantation of America, covering near vision spectacle independence, visual disturbance symptoms and patients satisfaction. It was determined whether patients have difficulties in near-distance reading (including computer using, and reading normal characters in newspaper and dispensatory) after surgery, and whether they need to wear glasses at the time of evaluating their near vision spectacle independence. As for visual disturbance symptoms, both glare and halo were included. Moreover, patient’s satisfaction with IOL was divided into “Very Satisfied”, “Satisfied”, “Generally Satisfied” and “Unsatisfied”; here, the total number of satisfactory eyes is equal to a sum of eyes with which the patients were very satisfied and satisfied.

### Statistical Analysis

Statistical analysis was performed with SPSS for Windows software (version 22, SPSS, Inc.). The Student’s t-test was used when the parameters followed a standard normal distribution. As for enumeration data, they are expressed in (n, %). Additionally, inter-group comparison was also carried out by means of χ^2^ test or Fisher’s exact test. The level of significance was a P value less than 0.05.

## RESULTS

### Postoperative visual acuity

After three months of the surgery, differences in UDVA, CDVA, DCIVA, DCNVA and CNVA of patients in both groups have no statistical significance (P>0.05). Nevertheless, both UIVA and UNVA of the SBL-3 group are statistically significantly superior to those of the SN6AD1 group (P>0.05), as shown in Table-II.

**Table-II:** Visual acuity 3 months postoperatively.

Parameter	MIOL SBL-3	SN6AD1	t	P value
UDVA (logMAR)	0. 02±0. 11	0. 03±0. 12	-0.315	0.754
CDVA (logMAR)	—0. 06±0. 07	—0. 05±0. 06	-0.56	0.578
UIVA (logMAR)	0. 14±0. 08	0. 21±0. 11	-2.621	0.011
DCIVA (logMAR)	0. 10±0. 09	0. 15±0.11	-1.798	0.078
UNVA (logMAR)	0. 15±0. 06	0. 20±0. 10	-2.234	0.031
DCNVA (logMAR)	0. 09±0. 07	0. 11±0. 10	-0.85	0.400
CNVA (logMAR)	0. 01±0. 08	0. 05±0. 09	-1.701	0.095

UDVA: Uncorrected distance visual acuity; CDVA: Corrected distance visual acuity; UIVA: Uncorrected intermediate visual acuity; DCIVA: Distance-corrected intermediate visual acuity; UNVA: Uncorrected near visual acuity; DCNVA: Distance-corrected near visual acuity; CNVA: Corrected near visual acuity.

### Postoperative contrast sensitivity

Table-III summarizes the contrast sensitivity of two groups at three months after surgery. The contrast sensitivity of the SBL-3 group outperforms the SN6AD1 group at four different spatial frequencies (3, 6, 12 and 18 cycles/degree) under mesopic conditions and mesopic conditions with glare. Their differences show statistical significance (P>0.05).

**Table-III:** Contrast sensitivity 3 months postoperatively.

Conditions	Group	Contrast sensitivity at diverse spatial frequencies			

		3c/d	6c/d	12c/d	18c/d
Mesopic conditions	SBL-3	1.48±0.15	1.52±0.18	1.19±0.15	0.62±0.32
	SN6AD1	1.28±0.13	1.31±0.20	0.93±0.18	0.36±0.22
	t	5.200	3.999	5.673	3.407
	*P* value	<0.001	<0.001	<0.001	0.001
Mesopic conditions with glare	SBL-3	1.26±0.13	1.29±0.21	0.89±0.20	0.38±0.27
	SN6AD1	1.14±0.11	1.16±0.25	0.72±0.23	0.25±0.18
	t	3.639	2.036	2.855	2.037
	*P* value	0.001	0.047	0.006	0.048

c/d: cycles/degree.

### Postoperative objective vision quality

Table-IV shows the ocular high order aberrations of the groups 3 months postoperatively. The total ocular high order aberration, coma and trefoil of SBL-3 group were statistically significantly higher than those of the SN6AD1 group (4 mm pupil diameter) (P<0.05). The differences of the groups showed no statistical significance in spherical aberrations (P>0.05). The MTF values of the groups three months postoperatively as shown in Table-V.. The MTF values of the SBL-3 group are all lower than those of the SN6AD1 group at spatial frequencies of 5, 10, 15, 20, 25 and 30 cycles/degree. Moreover, only differences in MTF values at 5, 10 and 15 cycles/degree were statistically significant (P<0.05).

**Table-IV:** Ocular high order aberrations 3 months postoperatively.

Aberrations	SBL-3	SN6AD1	t	P value
HOAs	0.39 ± 0.06	0.18 ± 0.07	11.654	<0.001
SA	0.03 ± 0.02	0.04 ± 0.02	-1.817	0.075
Coma	0.12 ± 0.04	0.09 ± 0.02	3.500	0.001
Trefoil	0.27 ± 0.09	0.11 ± 0.07	7.265	<0.001

HOAs: high-order aberrations; SA: spherical aberration.

**Table-V:** Modulation transfer function 3 months postoperatively.

Group	Modulation transfer function (MTF)					

	5c/d	10 c/d	15 c/d	20 c/d	25 c/d	30c/d
SBL-3	0.762±0.126	0.523±0.147	0.294±0.096	0.196±0.079	0.101±0.030	0.078±0.023
SN6AD1	0.875±0.082	0.637±0.138	0.367±0.123	0.241±0.105	0.121±0.057	0.092±0.046
t	-3.910	-2.911	-2.388	-1.746	-1.622	-1.423
*P* value	<0.001	0.005	0.021	0.087	0.112	0.162

c/d: cycles/degree.

### Postoperative questionnaire survey

After three months of the surgery, only one case from the SBL-3 group and two cases from the SN6AD1 group need to wear spectacles occasionally and the postoperative spectacle independence is figured out to be 96% (24/25) and 93% (26/28) respectively. Neither differences in spectacle independence between 2 groups (χ2=0.244; P=1.0) nor those in halo/glare incidence rates (4% & 1/25 vs.7% & 2/28, respectively) with χ2=0.014 and P=0.907 were of statistical significance. Regarding patient satisfaction (96% & 24/25 vs. 93% & 26/28), no statistical significance differences were found (χ2=0.244; P=1.0).

## DISCUSSIONS

The aim of multifocal intraocular lens (MIOL) use is to restore distance, intermediate and near visual function after cataract surgery.^5^ At present, there are two types of MIOLs in today’s clinical practice: the rotationally symmetric MIOL and a new concept of rotational asymmetric MIOL. Numerous studies have confirmed the efficacy to restore the visual function with rotationally symmetric MIOLs.^6^,^7^ However, optical side effects, such as decreased contrast sensitivity, glare, or halo, have also been reported frequently with these MIOLs.^8^ To reduce such side effects, refractive rotationally asymmetric MIOL has been introduced into clinical practice.

The Lentis Mplus (Oculentis GmbH, Berlin, Germany) was the first commercially available asymmetric MIOL, and several studies.^9^-^11^ have outlined the excellent vision achieved at various distances and a high level of patient satisfaction with reduced dysphotopsias and improved contrast sensitivity compared with some rotationally symmetric MIOLs. The SBL-3, a second-generation refractive rotationally asymmetric multifocal IOL, is based on the same principle of two refractive segments, but the near segment extends closer to the peripheral optic. Extended near segment could potentially result in fewer night vision optical disturbances and improved near vision. The SBL-3 has been clinically used to improve intermediate vision and reduce visual disturbances while restoring good visual outcomes at near and distance.^4^,^12^,^13^

In this study, patients from the SBL-3 group outperform those in the SN6AD1 group in UIVA and UNVA. Such an outcome is consistent with those reported by Wang. However, van der Linden et al.^15^ compared the Lentis Mplus with the Restor SN6AD1 IOL. They found that the IOLs achieved comparable distance vision, while the Restor provided better near visual acuity. McNeely et al.^12^ compared the Lentis Mplus with the SBL-3 IOL and found that the SBL-3 IOL provided better near visual performance than the Mplus IOL. The reason for this apparently better near vision with the SBL-3 IOL is not very clear. SBL-3 IOL has a larger surface area of near add without loss of the central aspect, the add reaches almost completely to the edge, and it has an equiconic biaspheric platform or some induction of aberration with this design, providing a larger depth of focus.^10^,^11^,^16^ These characteristics might contribute to its enhanced efficacy.

The reduction in contrast sensitivity is a major reason of dissatisfaction in patients with MIOL implantation.^8^ It is indicated in the present study that contrast sensitivity of patients in the SBL-3 group is statistically significantly superior to that of the SN6AD1 group at four diverse spatial frequencies under mesopic conditions and mesopic conditions with glare. Alio et al.^9^ compared the Lentis Mplus IOL with the Restor SN6AD3 IOL and found photopic contrast sensitivity was statistically significantly better with the Lentis Mplus IOL. With asymmetric optical sector design, more light is distributed to the distant focus. As only a little light loss is produced thanks to the existence of a transition zone, postoperative contrast sensitivity is high and close to the actual vision quality of normal subjects 20~60 years old.^17^

The total ocular high order aberration, coma and trefoil of SBL-3 group were statistically significantly higher than those of the SN6AD1 group. As for differences in spherical aberrations between both groups, no statistical significance is found (P>0.05). These outcomes conform to research findings reported by Wang et al.^14^ This suggests that implantation of a SBL-3 IOL can introduce greater high order aberrations. Through follow-up visits to patients with Lentis Mplus IOL implantation, high intraocular coma is proved among them. In opinions of researchers, the reason is the design of an optical zone of IOL.^10^,^11^ From perspectives of some other studies, it is deemed that not only does high order aberrations, such as coma and trefoil, of the implanted refractive segmental MIOL fail to preferably interpret postoperative vision quality of patients, but instrument measurement is subjected to a great influence of an additional optical sector zone. For these reasons, reference value of such studies is rather limited.^15^ However, posterior surface of the diffractive MIOL is designed to be a concentric diffraction ring, which causes a few disturbances to aberrometry and produces low coma correspondingly.

MTF describes a ratio of imaging contrast to image contrast at diverse spatial frequencies.^18^ It is under the influence of wave front aberrations and is able to objectively reflect optical imaging quality of the entire dioptric system in an IOL implanted eye. In the present study, MTF values of the SBL-3 group are all below those of the SN6AD1 group at diverse spatial frequencies. A previous study.^19^ indicated that HOAs may enhance the depth of focus while simultaneously lowering the MTF at higher frequencies. The introduction of a larger amount of intraocular aberrations may reduce retinal image quality of the eye implanted with SBL-3.

According to results of the questionnaire survey, near vision spectacle independence of SBL-3 and SN6AD1 groups is calculated to be 96% and 93% respectively. A study performed by McNeely et al.^12^ found that near vision spectacle independence is 93.3% for the SBL-3 group, which is similar to the results in our study. In the current study, there was no statistically significant difference in the incidence rate of halos and glare and patient satisfaction between the two groups. A Previous study.^20^ showed that 7.1% of the patients suffer severe halos, and 5.06% patients show obvious symptoms of glare after Lentis Mplus IOL implantation. In terms of the overall efficacy satisfaction, it is still rather high, reaching 97.5%. Clearly, these findings of our study are consistent with the research findings of Venter et al. In the design of the SBL-3 lens, loss of light in the transition between near and distance sector is negligible, and the lower the loss of energy with an IOL, the better contrast sensitivity and overall clarity of vision is expected.

## CONCLUSION

At three months after surgery, SBL-3 has the potential to provide satisfactory full range of vision and contrast sensitivity, improve spectacle independence of patients, induce fewer adverse postoperative visual symptoms and generate high patient satisfaction. In future studies, a larger sample size and more evaluation indexes should be provided on one hand; on the other hand, the follow-up period should be extended as well.

### Authors’ Contributions:

**Hl** **&** **WM:** designed this study and prepared this manuscript, & are responsible and accountable for the accuracy or integrity of the work;

**QW:** Collected and analyzed clinical data;

**XY:** significantly revised this manuscript.

## References

[1] Chang SW, Su TY, Chen YL (2015). Influence of ocular features and incision width on surgically induced astigmatism after cataract surgery. J Refract Surg.

[2] de Vries NE, Nuijts RM (2013). Multifocal intraocular lenses in cataract surgery:literature review of benefits and side effects. J Cataract Refract Surg.

[3] Portney V (2011). Light distribution in diffractive multifocal optics and its optimization. J Cataract Refract Surg.

[4] Venter JA, Barclay D, Pelouskova M, Bull CE (2014). Initial experience with a new refractive rotationally asymmetric multifocal intraocular lens. J Refract Surg.

[5] Gil-Cazorla R, Shah S, Naroo SA (2016). A review of the surgical options for the correction of presbyopia. Br J Ophthalmol.

[6] Alio JL, Elkady B, Ortiz D, Bernabeu G (2008). Clinical outcomes and intraocular optical quality of a diffractive multifocal intraocular lens with asymmetrical light distribution. J Cataract Refract Surg.

[7] Zelichowska B, Rekas M, Stankiewicz A, Cervino A, Montes-Mico R (2008). Apodized diffractive versus refractive multifocal intraocular lenses:optical and visual evaluation. J Cataract Refract Surg.

[8] Woodward MA, Randleman JB, Stulting RD (2009). Dissatisfaction after multifocal intraocular lens implantation. J Cataract Refract Surg.

[9] Alio JL, Plaza-Puche AB, Javaloy J, Ayala MJ (2012). Comparison of the visual and intraocular optical performance of a refractive multifocal IOL with rotational asymmetry and an apodized diffractive multifocal IOL. J Refract Surg.

[10] Ramon ML, Pinero DP, Perez-Cambrodi RJ (2012). Correlation of visual performance with quality of life and intraocular aberrometric profile in patients implanted with rotationally asymmetric multifocal IOLs. J Refract Surg.

[11] Alio JL, Pinero DP, Plaza-Puche AB, Chan MJ (2011). Visual outcomes and optical performance of a monofocal intraocular lens and a new-generation multifocal intraocular lens. J Cataract Refract Surg.

[12] McNeely RN, Pazo E, Spence A, Richoz O, Nesbit MA, Moore TCB (2017). Visual quality and performance comparison between 2 refractive rotationally asymmetric multifocal intraocular lenses. J Cataract Refract Surg.

[13] McNeely RN, Pazo E, Spence A, Richoz O, Nesbit MA, Moore TCB (2017). Visual outcomes and patient satisfaction 3 and 12 months after implantation of a refractive rotationally asymmetric multifocal intraocular lens. J Cataract Refract Surg.

[14] Wang X, Tu H, Wang Y (2020). Comparative Analysis of Visual Performance and Optical Quality with a Rotationally Asymmetric Multifocal Intraocular Lens and an Apodized Diffractive Multifocal Intraocular Lens. J Ophthalmol.

[15] van der Linden JW, van Velthoven M, van der Meulen I, Nieuwendaal C, Mourits M, Lapid-Gortzak R (2012). Comparison of a new-generation sectorial addition multifocal intraocular lens and a diffractive apodized multifocal intraocular lens. J Cataract Refract Surg.

[16] Alio JL, Plaza-Puche AB, Javaloy J, Ayala MJ, Vega-Estrada A (2013). Clinical and optical intraocular performance of rotationally asymmetric multifocal IOL plate-haptic design versus C-loop haptic design. J Refract Surg.

[17] van der Linden JW, Vrijman V, Al-Saady R, van der Meulen IJ, Mourits MP, Lapid-Gortzak R (2014). Autorefraction versus subjective refraction in a radially asymmetric multifocal intraocular lens [published correction appears in Acta Ophthalmol 2015; 93;(1): e82. El-Saady, Rana [corrected to Al-Saady, Rana]. Acta Ophthalmol.

[18] Diaz-Douton F, Benito A, Pujol J, Arjona M, Guell JL, Artal P Comparison of the retinal image quality with a Hartmann-Shack wavefront sensor and a double-pass instrument. Invest Ophthalmol Vis Sci. 2006;.

[19] Nio YK, Jansonius NM, Fidler V, Geraghty E, Norrby S, Kooijman AC Spherical and irregular aberrations are important for the optimal performance of the human eye. Ophthalmic Physiol Opt. 2002;.

[20] Venter JA, Pelouskova M, Collins BM, Schallhorn SC, Hannan SJ Visual outcomes and patient satisfaction in 9366 eyes using a refractive segmented multifocal intraocular lens. J Cataract Refract Surg. 2013;.

